# Fluorescence-Sensor Mapping for the in Vineyard Non-Destructive Assessment of Crimson Seedless Table Grape Quality

**DOI:** 10.3390/s20040983

**Published:** 2020-02-12

**Authors:** Lorenza Tuccio, Lucia Cavigli, Francesca Rossi, Olga Dichala, Fotis Katsogiannos, Ilias Kalfas, Giovanni Agati

**Affiliations:** 1 Istituto di Fisica Applicata “Nello Carrara” IFAC, Consiglio Nazionale delle Ricerche, Via Madonna del Piano 10, 50019 Sesto Fiorentino, Italy; l.tuccio@ifac.cnr.it (L.T.); l.cavigli@ifac.cnr.it (L.C.); f.rossi@ifac.cnr.it (F.R.); 2 American Farm School, 54 Marinou Antypa Street, P.O. Box 23, 55102 Thessaloniki, Greece; odicha@afs.edu.gr (O.D.); fkatso@afs.edu.gr (F.K.); ikalfa@afs.edu.gr (I.K.)

**Keywords:** anthocyanin mapping, chlorophyll mapping, Crimson Seedless, fluorescence, precision viticulture, optical sensors, table grape, zoning

## Abstract

Non-destructive tools for the in situ evaluation of vine fruit quality and vineyard management can improve the market value of table grape. We proposed a new approach based on a portable fluorescence sensor to map the ripening level of Crimson Seedless table grape in five different plots in the East, Central-North and South of the Macedonia Region of Greece. The sensor provided indices of ripening and color such as SFR_R_ and ANTH_RG_ correlated to the chlorophyll and anthocyanin berry contents, respectively. The mean ANTH_RG_ index was significantly different among all the plots examined due to the occurrence of different environmental conditions and/or asynchronous ripening processes. The indices presented moderate, poor in some cases, spatial variability, probably due to a significant vine-to-vine, intra-vine and intra-bunch variability. The cluster analysis was applied to the plot with the most evident spatial structure (at Kilkis). Krigged maps of the SFR_R_, ANTH_RG_ and yield were classified by k-means clustering in two-zones that differed significantly in their mean values. ANTH_RG_ and SFR_R_ were inversely correlated over 64% of the plot. SFR_R_ appeared to be a potential useful proxy of yield since it was directly correlated to yield over 66% of the plot. The grape color (ANTH_RG_) was slightly higher over the low-yield zones with respect to the high-yield zones. Our study showed that the combination of anthocyanins and chlorophyll indices detected in the field on Crimson Seedless table grape by a portable fluorescence sensor can help in defining the best harvest time and the best areas for harvesting.

## 1. Introduction

Precision agriculture techniques are rapidly expanding along with the fast digitalization of any life sector [1]. The market requirement of increasing quantity of high-quality fruit for long period of time asks for more precise tools to control quality and forecast product availability [2]. This is the case of table grape that is largely cultivated all over the world [3].

An objective control of ripening to define the best time of harvest is therefore of large interest. The ratio between sugar content and acidity seems to be the parameter that fits at the best the consumer preference for table grape [4]. Color and firmness are also important in determining the consumer choice of all fruits in general.

Quality traits of fruits are usually determined by destructive analyses that are time consuming and costly, as well as performed on a limited number of samples that are not representative of the whole crop. An early detection of quality in the field would be useful to manage the postharvest processing of table grape from selection and storability to distribution to the market. For this reason, attention has been paid to developing and applying photonics techniques for the non-destructive assessment of quality parameters in fruits.

Cavallo et al. [5] presented a computer vision system able to achieve an accurate classification of marketable white table grape. In lab hyperspectral imaging was applied to estimate titratable acidity, pH and sugar content of several table grape cultivars [6]. 3D-imaging approaches for the automatic determination of grape yield parameters [7] and grape bunch architecture [8] are under development. Anastasiou et al. [9] investigated the correlation between satellite and proximal reflectance sensing and yield and quality of Thompson Seedless table grape.

Fluorescence sensors has been largely employed in the wine grape sector to predict the phenolic maturity in the field [10]. 

Bahar et al. [11] applied for the first time the Multiplex fluorescence sensor to study ripening of Thompson Seedless and Crimson Seedless table grapes. The same sensor was employed to monitor non-destructively the accumulation of anthocyanins in Flame Seedless grapes because of defoliation and abscisic acid treatments [12].

Beside measuring with time a limited number of selected bunches, useful information can be obtained by mapping a whole vineyard. It is well known that vineyards may present significant spatial variability in both grape yield and quality [13], due to the soil characteristics and related water availability [14]. Quantification of this spatial variability by mapping can be used by adopting zone management practices to homogenize the vineyard or employed to perform temporal selective harvesting of bunches with the highest quality. Pothen and Nuske [15] recently introduced for the first time this approach on table grape cultivation by using a color image analysis.

Mapping bunch ripening by proximal fluorescence sensing was proved to be a useful tool for selective harvesting in precision viticulture [10,16,17]. Application of this technique to table grape, although needed as competitive advantage for the trade of the crop, is still lacking. 

The aim of this work is to introduce a new, non-destructive tool for the infield assessment of table grape maturity and its spatial variability. The application of this precision viticulture approach to the table grape cultivation can introduce zonal management of the vineyards with useful revenues in term of grape quality and productivity. It can be of large interest for the whole distribution chain from producers to importers and retailers.

## 2. Materials and Methods

### 2.1. Experimental Sites and Plant Management

The study was performed during the first week of September 2018 on Crimson Seedless (*Vitis vinifera* L.) table grape from vines cultivated at commercial vineyards in three different sites, Kavala, Katerini and Kilkis, in the East, South and Central-North, respectively, of the Macedonia Region (Greece). General information on the plots are reported in Table 1.

The shoots were trained to 8–10 buds with ten shoots per vine. For all plots watering was managed by drip irrigation. Standard cultural practices of plant growth regulators and fertilizers were used. The climatic conditions concerning air temperature, relative humidity and rainfall recorded in Katerini, Kavala and Kilkis during the 2018 season are reported as online Supplementary Materials (Figure S1).

### 2.2. Fluorescence Indices

The Multiplex® (Mx) fluorimetric sensor (FORCE-A, Orsay, France) is described in detail elsewhere [18]. Pictures on the use of the Mx in the field and additional information on the sensor are reported in Figure S2 of the online Supplementary Materials. The Mx measures fluorescence emitted by chlorophyll in the 670–690 nm red (RF) and 720–780 nm far-red (FRF) spectral regions, under excitation with four different light-emitting diodes (LEDs) at 375 nm (UV), 450 nm, 515 nm (G) and 630 nm (R). A third fluorescence channel with a yellow or a blue-green filter is available for further applications. Detection of fluorescence signals occurs through filtered photodiodes. The sensor is insensitive to ambient light, due to the use of pulsed excitation at 476 Hz with 20 μs per flash and synchronized detection so that it can be used directly in the vineyard. Each measurement consisted of a train of 250 flashes for each of the four excitation bands (UV, B, G and R). The mean and standard deviation of the 250 measurements for the 12 signals are visible on a real-time display and stored on a secure digital card, as csv files, for further elaboration.

Infield measured points are georeferenced by using an internal GPS. The area of detection is defined by a black mask with variable opening, up to 8-cm diameter, according to the size of the samples. Each sample is measured within an acquisition time less than 1 s. 

The basis of the fluorescence method applied by the Mx sensor is well described in Agati et al., 2013. Briefly, the intensity of the chlorophyll fluorescence (ChlF) emitted by a grape berry depends on the amount of excitation light able to reach the chlorophyll (Chl) pigment present inside the chloroplasts of the berry cells. The berry skin cell layers containing anthocyanins (Anth), localized above the Chl-containing cell layers, attenuate part of the incident light before this can reach the Chl molecules. Consequently, the higher the Anth concentration, the lower the ChlF intensity. The extent of the Anth attenuation also depends on the spectral band of the excitation light. Anth absorption extends from the green with maximum around 520 nm till red, therefore, their effect of attenuation of excitation can be observed at both G and R lights but at a different extent due to the shape of the Anth absorption spectrum.

Two Anth indices that are proportional to the berry skin Anth content can be defined as:

ANTH_RG_ = log (FRF_R_/FRF_G_), (1)

ANTH_R_ = log(1/FRF_R_), (2)

where FRF_R_ and FRF_G_ are the far-red ChlF signals excited by red and green light, respectively.

The first one, ANTH_RG_ (ANTH_RG, as reported in the Mx), is based on the differential absorbance of Anth between 515 and 630 nm, being a ratio, it is independent of the distance and the size of the sample measured. The second one, ANTH_R_, is based on a single fluorescence signal excited at 630 nm. This index is also named FERARI (Fluorescence Excitation Ratio Anthocyanin Relative Index) and reported as such, for “marketing” reasons, in the Mx sensor. However, we prefer to use the more appropriate nomenclature of Equation (2). The difference in using one or the other index on table grape is discussed in the next section.

The simple fluorescence ratio (SFR_R, as reported in the Mx),

SFR_R_ = FRF_R_/RF_R_, (3)

where RF_R_ is the red chlorophyll fluorescence signal excited by red light, is used as a Chl index, due to the partial reabsorption of RF by the Chl itself [19]. Further details on the origin of the above equations can be found in the literature [18,20,21].

### 2.3. Data Acquisition

At each vineyard, the optical sampling was based on a regular grid, the dimensions of which were determined by the vine and row spacing and the area of the plot. It was 11 by 12 m, 7.5 by 7 m, 7.5 by 6 m, 12 by 10 m and 21 by 18 m for Katerini, Kavala1, Kavala2, Kilkis1 and Kilkis2, respectively. At each grid point, four georeferenced clusters (two vines for each adjacent row) were manually measured by using the Mx sensor. The acquisition time varied between about 30 min and 1 h according to the plot size.

The yield of the Kilkis1 vineyard was evaluated by using a sampling grid of 3 × 4.8 m. At each grid point, the average weight of grape bunches per vine, harvested from pole to pole (4.8 m) along each row was considered.

### 2.4. Data Filtering

For each Mx measurement, the sensor records the mean and the SD over 250 40-µs flashes. The coefficient of variation due to accidentally sensor moving during the acquisition can then be calculated. Values of the FRF_R_ signal with a coefficient of variation larger than 20% were eliminated from the data set. After filtering, signals were normalized to a fluorescence standard (Urban Blue plastic foil, FORCE-A), before calculation of indices.

### 2.5. Statistical Analysis

Non spatial data were analyzed by using the SigmaPlot for Windows 14.0 (Systat Software, Inc., San Jose, CA, USA), and statistical differences among means were evaluated by the all-pairwise, multiple-comparison Holm–Sidak ANOVA test. P values of <0.05 were considered statistically significant.

### 2.6. Geostatistical Analysis and Mapping

The geostatistical analysis of measured data was performed to generate a continuous surface using Surfer version 11.0.642 (Golden Software, Inc.) and OriginPro 2015 (OriginLab Corporation) software. For each data set, different variogram models (spherical, gaussian, exponential) were tested by using a lag width similar to the sampling grid of each vineyard. The omnidirectional standardized variograms were used for the spatial interpolation by ordinary kriging and the results evaluated according to the mean squared error (MSE) [22] and the rank correlation at validation points (RCVP), between measurements and estimates, of the Surfer Cross Validation Report. The strength of the spatial dependence between measurements was evaluated by the Cambardella Index (CI) [23], defined as the ratio of the nugget variance to the sill. The mean correlation distance (MCD) [24] was used to estimate the distance over which the data have a high spatial dependence.

The interpolated maps were reported on a common color scale of equal intervals.

In order to compare the spatial variability of SFR_R_, ANTH_RG_ and yield of the Kilkis1 plot, the variograms for all attributes were fitted with a common set of input parameters (max lag distance = 22 m, number of lags = 25 and lag width = 11 m) and a spherical model. For each parameter, the resulting kriged maps were classified on the basis of k-means cluster analysis using two components.

For each pair of parameters, the single-variable maps were reclassified into four zones corresponding to the different combination of values, that is high–high (HH), low–low (LL), high–low (HL) and low–high (LH).

## 3. Results and Discussion

### 3.1. Plot Comparison

Prediction of phytochemicals by non-destructive methods requires the definition of a correlation curve between the indices and the compound concentration. It has been previously shown that both the ANTH_R_ and ANTH_RG_ indices provided by the Mx sensor are well correlated to the anthocyanins content of wine grape berries [16,17,25,26]. However, what is the best between the two Mx indices to be used for the prediction of Anth in grape is still under debate. Pinelli et al. [26] compared the Anth prediction performance of ANTH_R_ and ANTH_RG_ models as function of cultivar, vine growing site and season. It resulted that over the same season ANTH_RG_ was less accurate than ANTH_R_, with maximal relative errors of about 19% versus 14% in Anth prediction. On the other hand, ANTH_RG_ was more stable with years than ANTH_R_. The most appropriate Anth index depends also on the range of Anth concentration expected, since ANTH_R_ monotonically increases with Anth, while ANTH_RG_ shows a biphasic behavior, first increasing with Anth and then, at complete veraison, it decreases with Anth [18,26]. Therefore, while for ANTH_R_ a single correlation curve over the whole range of Anth can be used, two separate relationships must be applied for ANTH_RG_ according to the range of Anth. An upper limit of 160 µg cm^−2^ for Anth, below which the index shows a positive response to the anthocyanin content, was previously defined [18]. In the Crimson Seedless table grape, the Anth concentration is much lower than that found even in the low-accumulating wine grape varieties, such as Pinot Noir. Peppi et al. (2007) found that the Anth concentration in Crimson Seedless grape varied from 2.6 to 52.4 µg cm^−2^ depending on the season and the application of abscisic acid or ethephon treatments. 

Therefore, in this study, both Anth Mx indices should be valid without problems of ambiguity since both will be positively correlated to Anth, within the range of concentrations present in Crimson Seedless grapes. It must be, however, verified once the calibration curves for the Mx Anth indices applied to Crimson Seedless table grape will be available, as result of our scheduled future work. Nevertheless, application of the Mx sensor to follow ripening in the Malvasia Rosa variety, with pink colored berries, similarly to Crimson Seedless, showed a linear direct relationship between ANTH_RG_ and Anth all over the whole Anth range [27].

**Figure 1:**
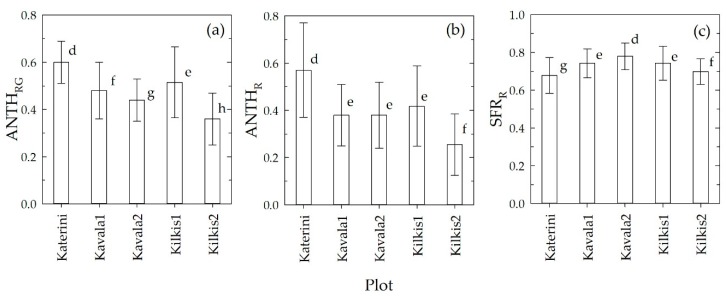
Mean (±SD) values of the ANTH_RG_ (**a**), ANTH_R_ (**b**) and SFR_R_ (**c**) Multiplex indices, measured in the Crimson Seedless table grape vineyard for each plot between 4 and 6 September, 2018. Within each graph, values with different letters are significantly different (P < 0.05) according to the Holm–Sidak test.

In Figure 1, the mean (±SD) values of the Mx indices recorded over each plot are reported. 

The mean ANTH_RG_ index was significantly different among all the plots examined, with the highest value observed in Katerini followed in sequence by Kilkis1, Kavala1, Kavala2 and Kilkis2. The highest and lowest content of Anth in Katerini and Kilkis2, respectively, was confirmed by ANTH_R_. However, ANTH_R_ was not able to discriminate the plots with intermediate levels of Anth. Kavala1 and Kilkis1 had the same mean values of SFR_R_, and they were significantly different that those of the other plots. Katerini reported the lowest level of SFR_R_, that is the lowest level of Chl, confirming that the grape at this site was the ripest.

The highest value of Anth predicted in Katerini grape can be explained as due to the lower temperature recorded at this site with respect to the other locations in the Aug–Sep period (Figure S1). In fact, it is well known that high temperatures decrease the anthocyanin content of berries through the reduction of the endogenous abscisic acid that is involved in the anthocyanin biosynthesis in grapes [28]. The effect was found to be particularly effective between one and three weeks after veraison [28]. On the other hand, Katerini experienced a number of degree days above 30 °C equal to 67 that was lower than Kilkis1, Kavala and Kilkis2, for which that number was 68, 77 and 78, respectively. Curiously, the increasing order of degree days above 30 °C corresponded to the decreasing order of ANTH_RG_ among the plots.

Temperature was seen to affect the skin color in several table grape varieties [29,30], included Crimson Seedless [31]. The diverse environmental conditions at each site may have dissimilarly affected grape ripening [32], so that the different values in Anth estimated on the plots at the beginning of September may be due to faster or slower rates of maturity. Accordingly, the date of veraison of Katerini grape (middle of July) occurred 10 days before that of Kilkis1.

### 3.2. Spatial Variability

The spatial variability of grape ripening was evaluated for the different plots by mapping the SFR_R_ and ANTH_RG_ indices. The first indicates the berry content of Chl that has been observed to be inversely correlated to the berry sugar content [10]. The second index is considered a proxy of the Anth berry level. The SFR_R_ index measured at veraison was also found to be directly well correlated to vine yield [25].

Generally, the variation of the ANTH_RG_ was higher than that of SFR_R_, according to the values of the coefficient of variation (CV) and spread (Table 2). Kilkis2 and Kavala2 showed the least variability in SFR_R_ with spread around 50%. The lowest and highest variability in ANTH_RG_ were observed in Katerini and Kilkis2, respectively. This indication suggests an advanced state of grape ripening in Katerini with respect to the other plots, in accordance with previous evidences that the variability of the Anth content in berries decreases from veraison to harvest [25].

The results of the geostatistical analysis of data are summarized in Table 2 and the relative variograms are reported in Figures S3 and S4, for SFR_R_ and ANTH_RG_, respectively, of the online Supplementary Materials. For most of the cases, the best fitting models (lowest MSE, highest RCVP) resulted to be the exponential with a nugget component. For Kavala1 and Kilkis2 the model to fit SFR_R_ data was linear.

It is clear that the SFR_R_ Chl index in Kavala1 and Kilkis2 did not show any spatial variability (CI = 100, MCD = 0), due to a very high nugget effect. Variograms resulted flat and almost flat, respectively (Figure S3b,e). For both Mx indices in Kavala2, the CI was close to the limit of weak spatial dependency and reported scarce scores in the cross validation.

**Figure 2:**
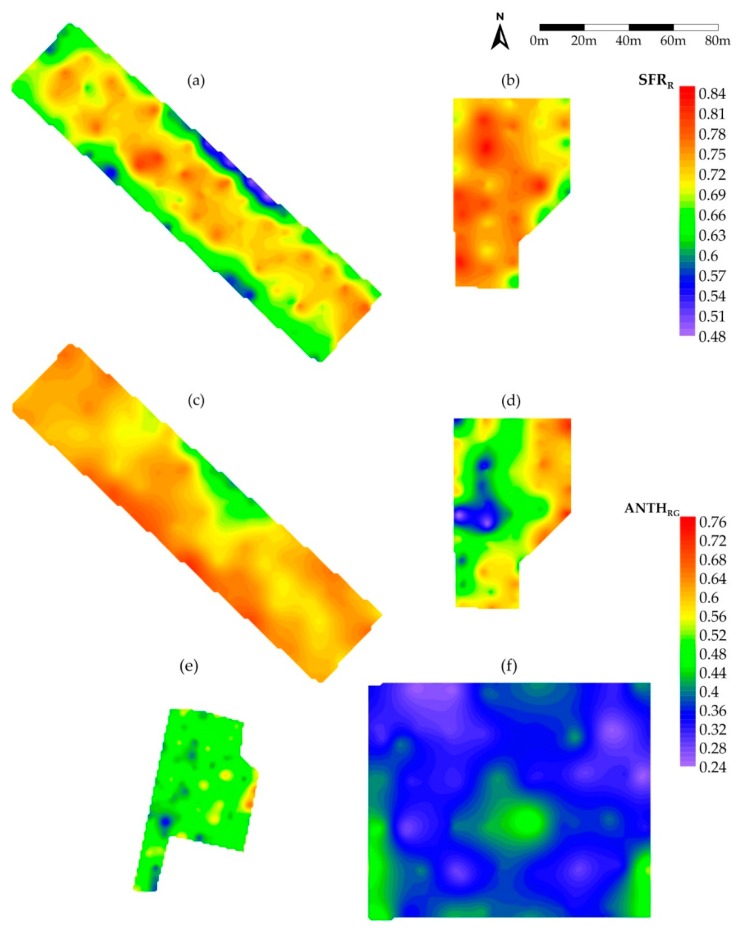
Krigged maps for the SFR_R_ (**a**,**b**) and ANTH_RG_ (**c**–**f**) indices measured in the Katerini (**a**,**c**), Kilkis1 (**b**,**d**), Kavala1 (**e**) and Kilkis2 (**f**) plots. For each index, maps are reported on a common color scale of equal intervals.

For all the other cases, SFR_R_ at Katerini and Kilkis1 as well as ANTH_RG_ at Katerini, Kavala1 and both Kilkis, the spatial dependence can be considered moderate (25 < CI < 75). The maps of these Mx indices that showed moderate spatial variability are reported in Figure 2a–b and Figure 2c–f for SFR_R_ and ANTH_RG_, respectively.

The small MCD values, below the threshold of the sampling grid, and the large nugget effect found for ANTH_RG_ can be explained as due to a great vine-to-vine, intra-vine, and intra-bunch variability of Anth [33].

The SFR_R_ in Katerini was higher in the inner rows of the plot and decreased towards the edge of it. In Kilkis1, SFR_R_ was almost spatially homogeneous with lower values on the East part and the South-East corner of the plot. The visual comparison of the two SFR_R_ maps confirms the higher content in Chl in Kilkis1 berries with respect to Katerini ones indicated by the average values reported in Figure 1, showing a delayed ripening process in the first site. This means, according to the inverse relationship existing between SFR_R_ and total soluble solids (°Brix) of berries, that the grape in Kilkis1 had a lower level of sugar with respect to Katerini grape. 

Large differences appeared in the spatial distribution of the ANTH_RG_ index among the plots (Figure 2c–e). In Katerini, the South-West border was the most reddish with the maximum Anth content, while minimum values were located at the middle of the North-East border. 

The Kilkis1 ANTH_RG_ showed a marked spatial structure, with the highest values at the East side of the vineyard and a minimal hollow in the central-West part of the plot. It is worth noting that although Kilkis1 and Kavala1 reported a rather similar average value of ANTH_RG_ (0.51 and 0.48, respectively, Figure 1), their spatial distribution of Anth was strikingly different. 

The Kilkis2 plot presented an almost homogeneous Anth spatial distribution with data around the lowest average ANTH_RG_ level (0.36) recorded among the plots.

The origin of the spatial structure of SFR_R_ and ANTH_R_ can be ascribed to the complex interaction of several parameters such as soil, microclimate and plant structures.

In Katerini, an evident ‘border effect’ likely due to increased sun exposure was observed. This could favor vine photosynthesis and the maturation process, but also cause Anth degradation as seen on the middle-East side. The less competition for resources among periferic plants with respect to inner vineyard plants could also affect grape ripening.

The same image more or less appears in Kilkis1, but with additional contribution of variability at the soil level, observed in the inner part of the plot, influencing the vine vigor and the maturation process of bunches.

### 3.3. Zoning

Zoning by classification and clustering of data can produce maps usable for the vineyard management and selective harvest. We applied the cluster analysis to the Kilkis1 plot that was that with the most structured spatial variability. At first, the interpolated maps of SFR_R_, ANTH_RG_ and yield were built by using variograms with common parameters and a spherical model for fitting, as reported in Figure S5. 

**Figure 3:**
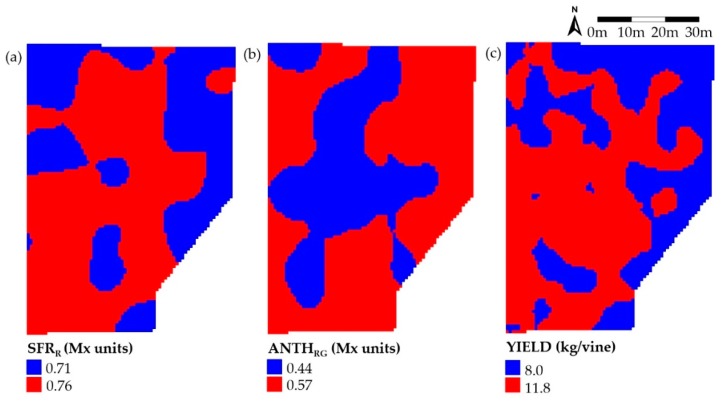
K-means clustering of the SFR_R_ (**a**) and ANTH_RG_ (**b**) Multiplex indices measured in the Kilkis1 plot and of the yield of the same plot (**c**) using two components. Values in the legend of each map represent the centroid of the clusters.

The krigged maps of the SFR_R_ and ANTH_RG_ were then classified by k-means clustering with two components, since two-class zoning is considered useful for practical running of selective harvesting. The zoned maps of the Mx indices are reported in Figure 3 along with the yield map determined on the same plot.

The pattern of SFR_R_ seems to be complementary to the ANTH_RG_ one. This was expected since the Chl and Anth content of berries decreases and increases with ripening, respectively. The SFR_R_ map was similar to the yield map with higher values in the central and South-West part of the plot. This is in accordance with previous results reported for the Tempranillo wine grape [25].

Usually, areas of poor yield are characterized by grape with higher concentrations of colour (Anth). This trend was confirmed here, as the central part of the plot showed lower ANTH_RG_ values and higher yield and the opposite was observed on the East edge of the plot.

The values of the cluster centroids reported in the legend of Figure 3a–c does not allow to know if the two zones are significantly different to have an impact on separate management. For this, the zone-based means for the various grape parameters were calculated from the raw data of vines belonging to the two low- and high-value areas. 

These values resulted to be 0.68 and 0.80, 0.37 and 0.60, 5.7 and 13.6 kg/vine for SFR_R_, ANTH_RG_ and yield, respectively, and for each couple, they differed significantly (P < 0.001).

Pearson correlation coefficients confirmed the positive correlation between SFR_R_ and yield (r = 0.36) and the negative correlation between SFR_R_ and ANTH_RG_ (r = −0.62) and between ANTH_RG_ and yield (r = −0.37). All correlations were significant at P < 0.001. The correlation between SFR_R_ and yield was found to be weaker than that found in Tempranillo grape (r = 0.80) [25]. This may depend on the different period of detection of the Mx index that was at veraison for Tempranillo and close to harvest for Crimson Seedless in the present study.

Actually, the correlation between yield and SFR_R_ can derive from the combination of two contributions. There is a direct contribution considering SFR_R_ as index of Chl in the berries, that is, higher SFR_R_ means higher vine vigor and higher yield. On the other hand, after veraison the decrease of SFR_R_ with time can be considered an index of ripening that is inversely related to the berry total soluble solids (Brix) [10,11]. Since often a negative relationship exists between Brix in berries and grape yield [34,35], indirectly, low values of SFR_R_ at harvest may correspond to low yields.

**Figure 4:**
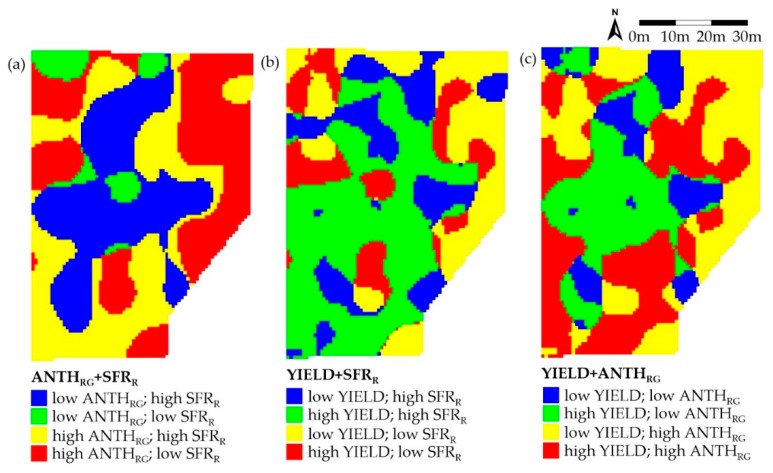
Combined maps for the couple of ANTH_RG_+SFR_R_ (**a**), Yield+SFR_F_ (**b**) and Yield+ANTH_RG_ (**c**) variables.

The spatial relationship between variables can be better evaluated by the combined maps reported in Figure 4. 

For the ANTH_RG_+SFR_R_ combination, the zones with complementary values (blue and red colored) were predominant. For the yield+SFR_R_ pair, a large area with higher values of both parameters (green) was covered. For the yield+ANTH_RG_ pair, the low-low combination (blue) was the less represented while the other three combinations were similar.

In Table 3, the percentage of areas covered by the different combinations of parameters in the maps of Figure 4 are reported. This analysis confirms that ANTH_RG_ and SFR_R_ are mainly inversely related over the plot, since the total area covered by the sum of high + low (HL) and low + high (LH) values of the indices amounts to about 64%. Table 3 shows also that SFR_R_ and yield are mostly correlated, since the total area covered by the sum of their HH and LL values amounts to almost 66%. The inverse correlation between ANTH_RG_ and yield is only partially confirmed by this evaluation, since the total area covered by the sum of their HL and LH values amounts to 54.4%.

It is interesting to note that the most appealing area of high grape color (high ANTH_RG_) and high yield derived from Figure 4c (33.2%, red colored) is rather similar to that represented by the high-ANTH_RG_ plus high-SFR_R_ values in the Figure 4a map (29.6%, yellow colored). 

This evidence further induces to evaluate the SFR_R_ index as a proxy of grape yield, as complementary or even alternative tool to satellite or proximal reflectance sensing indices of vine vigor [9].

## 4. Conclusions

The present study proved the utility of the Chl fluorescence-based technology and the associated geostatistical analysis to evaluate non destructively in situ the quality of Crimson Seedless table grape. The technology can be also applied to white table grape for which the flavonol index (FLAV) represents an additional quality parameter.

The relationship between SFR_R_ and yield should be better evaluated by monitoring the Mx index during the season. Its values before veraison, at full berry development, are expected to be correlated at the best to vine vigor and grape yield. 

The combination of the assessment of the Anth and Chl grape constituents under temporal and spatial monitoring can provide useful information for defining the best harvest time and the best areas for harvesting. Zonal management may offer advantages with respect to when vineyards are managed uniformly.

This would be of great interest for developing appropriate prediction models forecasting the amount of grape available at a defined quality standard at specific points in the field.

When this is achieved, the production of table grapes will be a more programmable activity with envisaged advantages for producers, importers and retailers. 

The results of the present research are promising and must be validated in future works on a more extended period of time during the season and over different seasons. They also open a new prospect for studying the correlation between the Mx indices and different table grape quality parameters, such as yield, bunch weight, sugar content and colour, to achieve a more complete characterization of this precision viticulture technology. These will be the matter of our future work along with the calibration of the non-destructive indices of Anth content against wet chemistry for the Crimson Seedless grape cultivar.

## Figures and Tables

**Table 1:** Characteristic parameters of the Crimson Seedless table grape vineyards investigated.

Name	Location (Latitude Longitude)	Mean Altitude (m a.s.l.)	Area (m^2^)	Planting Distance (m) ^1^	Row Orientation	Training System	Plant Age (Years)	Yield (2018) (tn ha^−1^)
Katerini	40°12′52.30″N 22°25′50.06″E	89	7900	3.6 × 2.2	NW–SE	Semi-pergola	11	16
Kavala1	40°49′30.88″N 24°16′22.55″E	15	2800	2.5 × 1.6	NNE–SSW	Y-shaped trellis	8	48
Kavala2	40°48′38.35″N 24°13′32.48″E	179	2100	2.5 × 1.6	NNW–SSE	Y-shaped trellis	6	45
Kilkis1	41° 3′11.64″N 22°57′32.02″E	257	4400	3.0 × 2.4	N–S	Y-shaped trellis	7	20
Kilkis2	41°4′41.83″N 22°46′40.58″E	102	14200	3.0 × 2.4	E–W	Pergola	10	35

^1^ Inter x intra rows.

**Table 2:** Descriptive statistics and geostatistical analysis results for the fluorescence-based SFR_R_ and ANTH_RG_ indices acquired in the different Crimson Seedless table grape vineyards of the Macedonia Region.

Index	Plot	Spread (%)	CV (%)	CI (%)	MCD (m)	MSE	RCVP
SFR_R_							
	Katerini	73	14.2	30.00	5.51	0.005	0.583
	Kavala1	70	10.3	100	0.00	--	--
	Kavala2	52	9.1	69.30	1.21	0.0046	0.099
	Kilkis1	62	12.7	52.53	3.04	0.007	0.452
	Kilkis2	49	9.7	100.00	0.00	0.005	0.199
ANTH_RG_							
	Katerini	99	15.5	52.88	6.63	0.006	0.449
	Kavala1	136	24.5	50.00	1.58	0.011	0.342
	Kavala2	99	20.4	74.29	1.45	0.008	0.158
	Kilkis1	152	29.5	30.34	5.33	0.014	0.604
	Kilkis2	157	31.7	53.59	6.27	0.009	0.584

**Table 3:** Percentage of areas covered for each combination of variables in the combined maps of Figure 4.

Map	Cluster Area (%)					
	High–High	Low–Low	Low–High	High–Low	HH + LL ^1^	HL + LH ^1^
ANTH_RG_+SFR_R_	29.6	6.3	31.5	32.7	35.8	64.2
Yield+SFR_R_	42.7	23.2	18.4	15.7	65.9	34.1
Yield+ANTH_RG_	33.2	12.4	29.1	25.3	45.6	54.4

^1^ H and L mean high and low values, respectively.

## Supplementary Materials

The following are available online at https://www.mdpi.com/1424-8220/20/4/983/s1. Figure S1: Meteorological conditions of air temperature (a), relative humidity (b) and rainfall (c) registered by means of weather stations close to the investigated sites. Maximal and minimal air temperature and relative humidity are the mean values over 15 days. Rainfall is the sum of precipitation over 15 days. Figure S2: Pictures showing the use of the Multiplex sensor in the field (a). Lateral and front view of the optical head (b). Figure S3: Experimental (open symbols) and fitted (solid lines) standardized variograms for the SFR_R_ data acquired in the Katerini (a), Kavala1 (b), Kavala2 (c), Kilkis1 (d) and Kilkis2 (e) plots. Figure S4: Experimental (open symbols) and fitted (solid lines) standardized variograms for the ANTH_RG_ data acquired in the Katerini (a), Kavala1 (b), Kavala2 (c), Kilkis1 (d) and Kilkis2 (e) plots. Figure S5: Experimental (open symbols) and fitted (solid lines) standardized variograms for the SFR_R_ (a), ANTH_RG_ (b) and yield (c) data acquired at the Kilkis1 plot. A spherical fitting function was used for each parameter. Table S1: Functions and parameters used for the variograms reported in Figure S3 for SFR_R_. Table S2: Function and parameters used for the variograms reported in Figure S4 for ANTH_RG_. Table S3: Function and parameters used for the variograms reported in Figure S5.
