# Supplementary material for: Fluorescence-Sensor Mapping for the in Vineyard Non-Destructive Assessment of Crimson Seedless Table Grape Quality
Source: Sensors (Basel). 2020 Feb 12;20(4):983. doi: 10.3390/s20040983 (PMC7070766; doi:10.3390/s20040983)
Supplement: Supplementary file 1 [file sensors-20-00983-s001.pdf]

Supplementary Materials

Fluorescence-sensor mapping for the in vineyard non-destructive assessment of Crimson Seedless table grape quality - Tuccio et al.

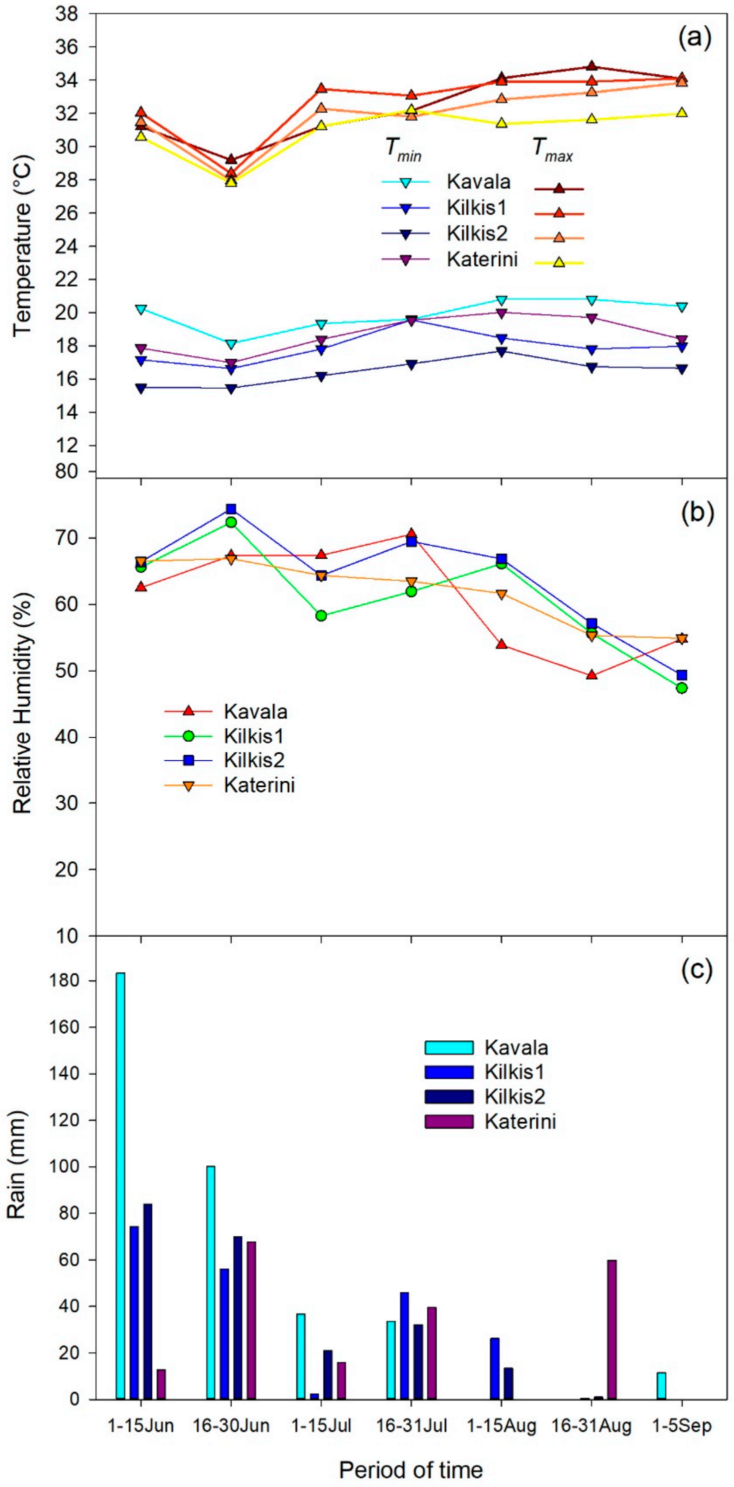

Figure S1. Meteorological conditions of air temperature (a), relative humidity (b) and rainfall (c) registered by means of weather stations close to the investigated sites. Maximal and minimal air temperature and relative humidity are the mean values over 15 days. Rainfall is the sum of precipitation over 15 days.

(a)

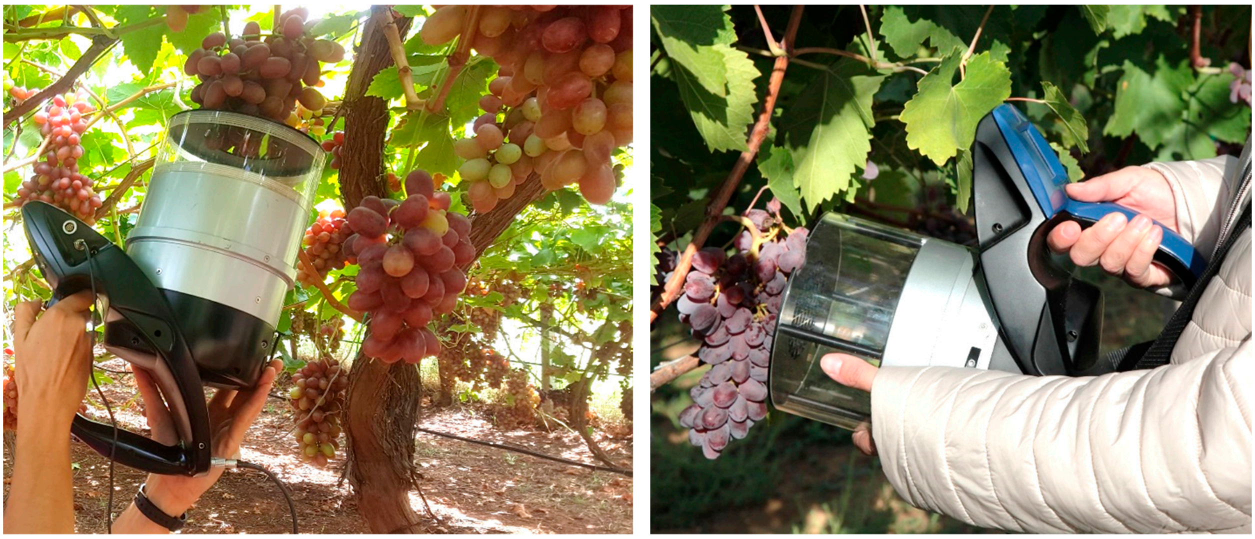

(b)

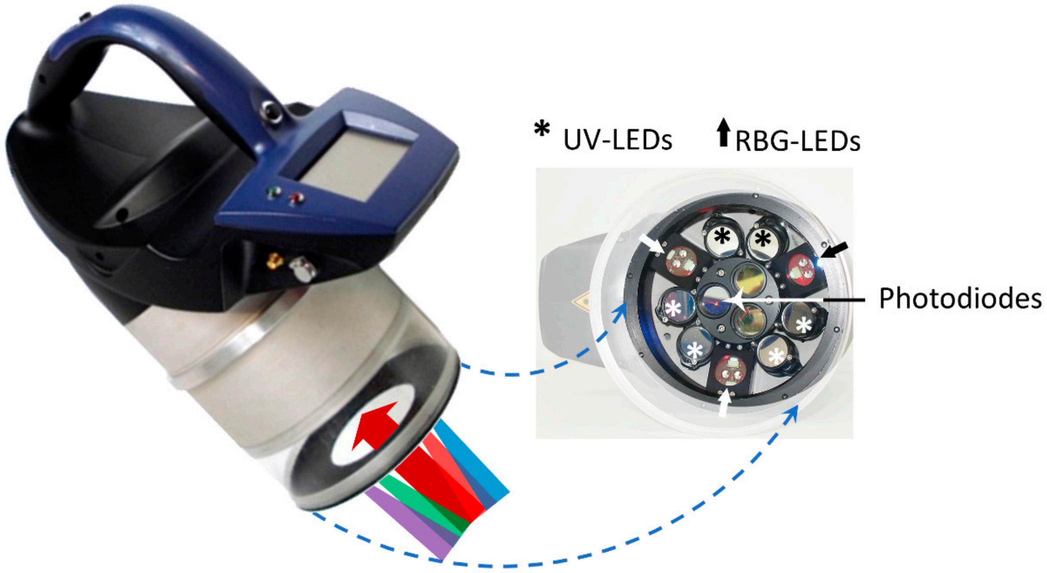

Figure S2. Pictures showing the use of the Multiplex sensor in the field (a). Lateral and front view of the optical head with LED sources in the external edge (6 UV* and 3 RGB^{arrows}) and three detectors in the middle (b). The six UV-LEDs emit at 375 nm and are protected by DUG11 filters (Schott, Mainz, Germany). The Red-Blue-Green (RGB) LED-matrices emitting lights at 470 nm, 515 nm and 630 nm protected by a 650-nm short-pass filter (Edmund Scientific, United Kingdom). LEDs are pulsed sequentially at 476 Hz with 20 μs per flash and are synchronised to three photodiode detectors for fluorescence recording in the yellow (around 590 nm), red (around 685 nm) and far-red (around 735 nm) spectral bands, which are defined by the 590NB10, 678WB22 and 750WB65 interference filters (Intor, Socorro, NM USA), respectively. In addition, the RF channel has a 3-mm RG665 red glass filter (Schott, Mainz, Germany) and the FRF channel has a 3-mm RG9 far-red glass filter (Schott, Mainz, Germany).

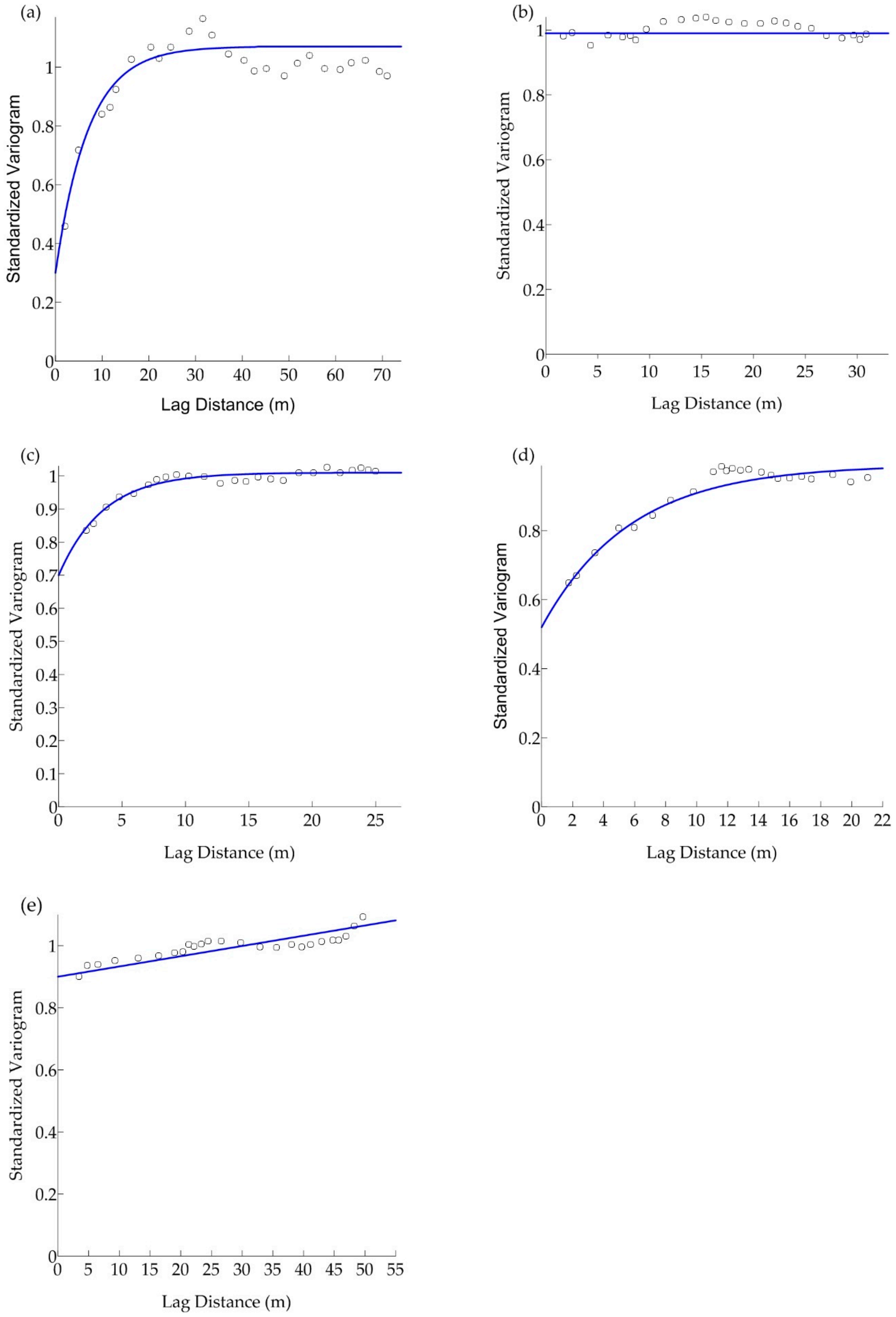

Figure S3. Experimental (open symbols) and fitted (solid lines) standardized variograms for the SFR_R data acquired in the Katerini (a), Kavala1 (b), Kavala2 (c), Kilkis1 (d) and Kilkis2 (e) plots. The fitting functions and parameters are reported in Table S1.

Table S1. Functions and parameters used for the variograms reported in Figure S3 for SFR_R.

Exponential model: $f(x) = c_0 + c_1\left[1 - e^{\left(-\frac{x}{A}\right)}\right]$, Linear model: $f(x) = c_0 + bx$

[1]where: c_0= nugget, c_1=scale, c_0+c_1= sill, A=length and a (range) = 3A; b =*slope*

**SFR_R**	**Plot**	***model***	c_0	c_1	A	b
	Katerini	exponential	0.3	0.77	7	
	Kavala1	linear	0.99	-	-	0
	Kavala2	exponential	0.7	0.31	3.5	
	Kilkis1	exponential	0.52	0.47	5.7	
	Kilkis2	linear	0.9	-	-	0.0033

[1] [1-3].

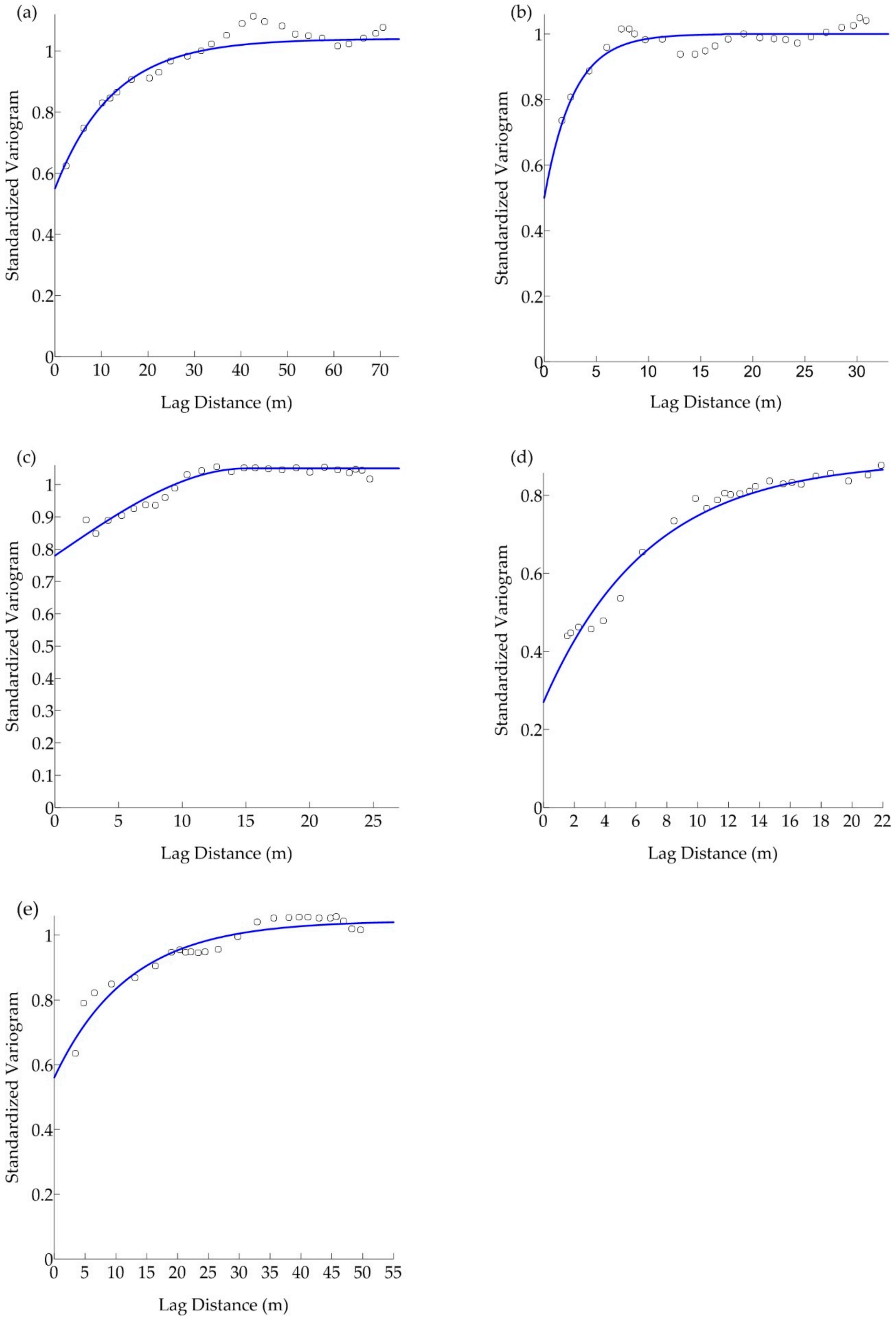

Figure S4: Experimental (open symbols) and fitted (solid lines) standardized variograms for the $ANTH_{RG}$ data acquired in the Katerini (a), Kavala1 (b), Kavala2 (c), Kilkis1 (d) and Kilkis2 (e) plots. The fitting functions and parameters are reported in Table S2.

Table S2. Function and parameters used for the variograms reported in Figure S4 for ANTH$_{RG}$.

All models were exponential: $f(x) = c_0 + c_1\left[1 - e^{\left(-\frac{x}{A}\right)}\right]$,

[1]where: c_0= nugget, c_1=scale, c_0+c_1= sill, A=length and a (range) = 3A

**ANTH$_{RG}$**	**Plot**	c_0	c_1	A
	Katerini	0.55	0.49	12.5
	Kavala1	0.5	0.5	2.8
	Kavala2	0.78	0.27	15
	Kilkis1	0.27	0.62	6.8
	Kilkis2	0.56	0.485	12

[1] [1-3].

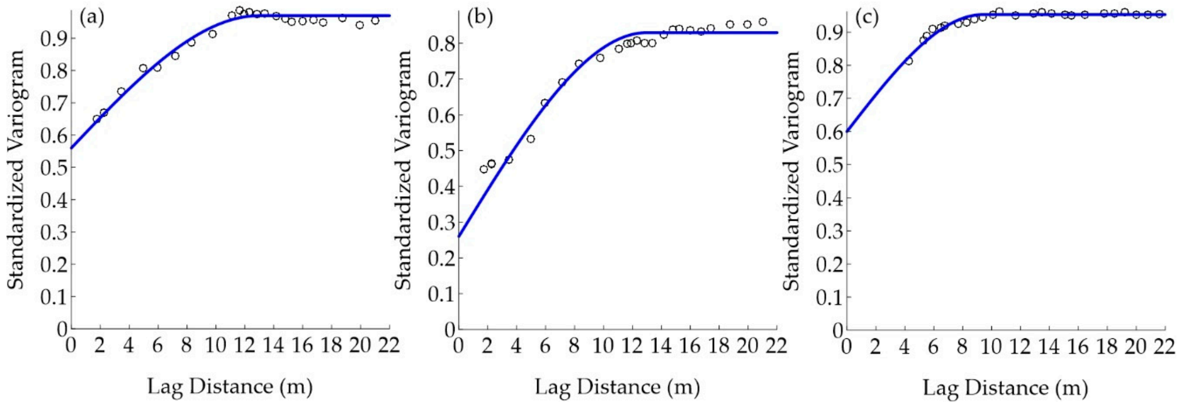

Figure S5. Experimental (open symbols) and fitted (solid lines) standardized variograms for the SFR$_R$ (a), ANTH$_{RG}$ (b) and yield (c) data acquired at the Kilkis1 plot. A spherical fitting function was used for each parameter. The fitting parameters are reported in Table S3.

Table S3. Function and parameters used for the variograms reported in Figure S5.

All models were spherical: $f(x) = c_0 + c_1\left[\frac{3x}{2A} - \frac{1}{2}\left(\frac{x}{A}\right)^3\right]$,

[1]where: c_0= nugget, c_1=scale, c_0+c_1= sill, A=length and a (range) = A

Kilkis1	Parameter	c_0	c_1	A
	SFR$_R$	0.56	0.41	13
	ANTH$_{RG}$	0.26	0.57	13
	Yield	0.6	0.353	9.4

[1] [1-3].

1. Cambardella, C.A.; Moorman, T.B.; Parkin, T.; Karlen, D.; Novak, J.; Turco, R.; Konopka, A., Field-scale variability of soil properties in central Iowa soils. *Soil Sci. Soc. Am. J.* **1994,** 58, 1501-1511.
2. Han, S.; Evans, R.G.; Schneider, S.M.; Rawlins, S.L., Spatial variability of soil properties on two center-pivot irrigated fields. *Precis. Agric.* **1996**, 97-106.
3. Oliver, M.; Webster, R., A tutorial guide to geostatistics: Computing and modelling variograms and kriging. *Catena* **2014,** 113, 56-69.
